# Quantitative assessment of synovitis in patients with rheumatoid arthritis using fluorescence optical imaging

**DOI:** 10.1186/ar4304

**Published:** 2013-09-18

**Authors:** Valentin S Schäfer, Wolfgang Hartung, Patrick Hoffstetter, Jörn Berger, Christian Stroszczynski, Martina Müller, Martin Fleck, Boris Ehrenstein

**Affiliations:** 1 Department of Rheumatology and Clinical Immunology, Asklepios Medical Center Bad Abbach, 93077 Bad Abbach, Germany; 2 Department of Radiology, Asklepios Medical Center Bad Abbach, Bad Abbach, Germany; 3 Mivenion GmbH, Berlin, Germany; 4 Department of Radiology, University Medical Center Regensburg, Regensburg, Germany; 5 Department of Internal Medicine (I), University Medical Center Regensburg, Regensburg, Germany

## Abstract

**Introduction:**

To prospectively evaluate quantitative assessment of fluorescence optical imaging (FOI) for differentiation of synovitic from non-synovitic joints in patients suffering from rheumatoid arthritis (RA).

**Methods:**

FOI of the hands was performed in patients with active RA, and a stratified quantitative fluorescence readout (FLRO) of 3 phases (1-120 s; 121-240 s; 241-360 s) was generated for 5 individual joints of the clinical predominant hand (carpal joint, metacarpophalangeal and proximal interphalangeal joints of digits II & III). To dissect the effect of the overall perfusion of the hand from the perfusion due to synovitis, a fluorescence ratio (FLRA) was additionally calculated, dividing each FLRO by the readout of the eponychium of digit II. The mean FLRO and FLRA were compared between joints with absent vs. present synovitis determined by clinical examination, grayscale, color Doppler ultrasonography, or magnetic resonance imaging (MRI).

**Results:**

The analysis for 90 individual joints from 18 patients yielded FLRO ranging from 4.4 to 49.0 × 10^3^, and FLRAs ranging from 0.37 to 2.27. Overall, the analyses based on the FLRA revealed a higher discrimination than the analyses related to the FLRO, demonstrating most significant differences in phases 2 and 3. A sensitivity of 26/39 (67%) and a specificity of 31/40 (77%) were calculated for FLRA of phase 3 using a cut-off value of more than 1.2 to detect MRI-confirmed synovitis with FOI.

**Conclusions:**

FOI has a potential for visualizing synovitis in subjects with RA. For adequate FOI interpretation, quantitative analysis should be based on the novel FLRA calculated for phases 2 and 3.

## Introduction

Rheumatoid arthritis (RA) is an inflammatory disorder involving multiple joints [1,2]. It is the most common chronic inflammatory joint disease with a prevalence of 0.5 to 1.0% [3,4]. Persistent synovitis leads to massive joint destruction, and eventually causes irreversible disability in patients. It is of uttermost importance to recognize early synovitis, before substantial joint damage occurs and to start early and aggressive therapy in order to improve short- and long-term outcomes [5-7]. Therefore, sensitive and specific tools for early diagnosis of RA are necessary [5,6]. The clinical examination (CE) is a prerequisite, but may miss subclinical inflammation in patients with early disease as well as in those who are in clinical remission under treatment [8]. Conventional x-ray examination is often used as an indicator of prognosis and represents the standard outcome measure of disease progression, but does not display the current disease activity [9]. Ultrasonography (US) is a valid tool in the assessment of patients with synovitis and in scoring the clinical activity [10-12]. US is, however, a time-consuming, and operator-dependent technique and might miss signs of early arthritis [13-15]. Magnetic resonance imaging (MRI) has proven to be the strongest independent predictor of radiographic progression in patients with RA [5,9]. MRI, however, is costly, time-consuming, and not ubiquitously available. For fast and dynamic assessment of joint inflammation, US is more widely available than MRI in daily clinical practice [16]. The disadvantage of US is that it is a time-consuming method, and, apart from in clinical studies, the examination is usually limited to a reduced number of joints due to time constraints.

Fluorescence optical imaging (FOI) is a new, non-invasive and non-ionizing imaging technology with fast acquisition times [17,18]. The major drawback of FOI is the limited tissue penetration of light; however, as inflammatory arthopathies typically affect the small joints of the hands and feet, this is not necessarily a significant limitation for this imaging method [19]. Under various experimental conditions, FOI proved to correspond to synovitis [17,18,20-24]. In those experiments, early hyperemia of inflamed joints could be diagnosed by recording scattering and absorption patterns of light transmitted through inflamed finger joints. This approach has already been tested in humans [21,25], which led to the development of an FOI system with fixed optical geometry (Xiralite X4; Mivenion GmbH, Berlin, Germany). The fluorescent dye indocyanine green (ICG) appears to be an appropriate tracer, because it has been shown to enhance inflamed joints [20,26]. Furthermore, this substance has been approved by the Federal Drugs Administration. In a study by Scheel et al. [27] FOI provided information about the inflammation status of finger joints with a sensitivity and specificity of 80% and 89%, respectively. In a clinical study by Werner et al. [8] FOI had, taking MRI as a reference, a sensitivity of 76% and a specificity of 54%. Recently, another study by Meier et al. [19] reported that FOI had a sensitivity of 39.6% and a specificity of 85.2%, compared with MRI demonstrating conflicting results regarding the reliability of this new method. In 2011 Dziekan et al. [28] proposed a quantitative analysis of FOI using normalized variances of fluorescence time correlation functions. In their pivotal pilot study, the joints of healthy volunteers and patients with RA were compared, but no other imaging techniques besides FOI were employed. Therefore, the purpose of this study was to establish a novel quantitative readout for ICG-enhanced FOI to allow more accurate joint assessment and to prospectively compare this imaging method with CE, US, and contrast enhanced 1.5 T MRI for detection of inflamed and non-inflamed joints of the hands in patients with proven RA.

## Methods

### Patients

Patients with known RA seeking routine clinical care and reporting symptoms suggestive of wrist and/or finger joint involvement were asked to participate in the study. Inclusion criteria were an established diagnosis of RA according to the American College of Rheumatology criteria from 1987, elevated rheumatoid factor (RF), and/or elevated anti-cyclic citrullinated peptide (anti-CCP) antibodies, and reported symptoms suggestive of wrist and/or finger-joint involvement. Exclusion criteria were pregnancy, renal failure, increased skin pigmentation [29], known allergy against iodine, ICG or gadolinium, or other contraindications for MRI. The study was performed in compliance with the Declaration of Helsinki. The study protocol was approved by the ethics committee of the University Medical Center Regensburg. All study participants signed consent forms after receiving appropriate written and oral information prior to enrollment.

Demographical data (sex, age, disease duration) and laboratory data (RF, anti-CCP, serum C reactive protein (CRP) and erythrocyte sedimentation rate (ESR)) were derived by chart review. All patients received a standardized rheumatologic CE of the 28 joints included in the Disease Activity Score 28 (DAS28, [30]) by one of the authors (V.S. or W.H.) using a bimanual technique, documenting tender and swollen status for each joint. The DAS28 was calculated for each patient [30].

All data regarding clinical and imaging studies were obtained within two days after inclusion for each enrolled patient.

### Ultrasonography

The joints included in the US7 score (wrist, metacarpophalangeal (MCP) and proximal interphalangeal (PIP) joints of the index (II) and middle (III) finger of the clinically dominant hand, and the metatarsophalangeal (MTP) joints of the second (II) and fifth (V) toe of the clinical predominant foot) were examined by ultrasonography by one of the authors (V.S. or W.H.) using a LOGIQ E9 (GE, Munich, Germany) ultrasound machine with a linear transducer (ML 6–15) with 15 MHz frequency [31]. Each joint was rated separately semiquantitatively (grades 0 to 3) for distension of the capsule due to synovitis with grey scale ultrasonography (GSUS) and for synovial vascularity with color Doppler ultrasonography (CDUS). The presence of synovitis was concluded for each mode if at least grade 1 findings were observed. For each patient the US7 score was calculated [31].

### Magnetic resonance imaging

For each patient a gadolinium-enhanced MRI of the clinically dominant hand was performed using a 1.5 T high-field MR scanner. The main focus of the MRI examination was the evaluation of the carpal and MCP joints, and if included in the examination field also the evaluation of the PIP joints. The examination protocol was performed analog to routine clinical practice and in line with the recommendations of the RAMRIS (Rheumatoid Arthritis MRI Scoring system) [32]. Fat saturated proton or T2-weighted and contrast-enhanced T1-weighted sequences were used in at least one coronal and axial plane. Additional unenhanced T1-weighted sequences were acquired in coronal plane. One of the 18 patients was examined with fat saturated proton-weighted and T2-weighted sequences, and the other patients were analyzed utilizing only proton-weighted images. For all patients the contrast-enhanced T1-weighted images were fat saturated using spectral saturating technique. Gadoteric acid (Dotarem^®^, Guerbet, Villepinte, France) was applied as intravenous contrast agent using a dosage of 0.2 ml/kg. (The majority of patients were examined using the following MRI protocol: prone position with the hand placed over the head in an extremity surface flex coil, coronal fat saturated proton-weighted turbo spin echo (Cor PD TSE fs), echo time (TE) 29/repetition time (TR) 2260, slice thickness (ST) 2.5 mm, gap 0.25 mm, field of view (FOV) 240 × 320 mm^2^, scan time: 1 minute 40 seconds. Sagittal fat saturated proton-weighted turbo spin echo (Sag PD TSE fs), TE 29/TR 3630, ST 3 mm, gap 0.3 mm, FOV 240 × 320 mm^2^, scan time: 1 minute 45 seconds. Coronal T1-weighted turbo spin echo (Cor T1 TSE), TE 14/TR 586, ST 2.5 mm, gap 0.25 mm, FOV 256 × 320 mm^2^, scan time: 2 minutes 16 seconds. Axial contrast-enhanced T1-weighted fat saturated turbo spin echo (Axial CE T1 TSE fs), TE 14/TR 732, ST 2.5 mm, gap 0.25 mm, FOV 236 × 192 mm^2^, scan time: 1 minute 40 seconds.) All MRI scans were read according to the RAMRIS scoring system by an experienced radiologist (P.H., seven years of MRI experience) blinded to clinical and ultrasonography data. The presence of synovitis was concluded for each joint, if at least grade one findings were observed. Additionally, the extensor tendons overlying the joint were evaluated for the presence of tenosynovitis. The RAMRIS score has been calculated for each patient.

### Fluorescence optical imaging

A commercially available, near-infrared fluorescence imaging system (Xiralite X4, Mivenion GmbH, Berlin, Germany) was used in this study. The working principle is based on the excitation of ICG dye by light emitting diodes and the detection of fluorescence signals with a sensitive camera. The instrument is controlled by a complementary PC and records image sequences with a standardized frame rate of one frame (image) per second over a period of six minutes. Thus, a stack of 360 images is provided for each individual examination.

The FOI examination followed a standardized procedure: both hands were placed on a preformed hand rest. Ten seconds after starting the examination, an ICG bolus (0.1 mg/kg) was injected manually in the cubital vein over the period of approximately one second (ICG-Pulsion, Pulsion, Feldkirchen, Germany). Any alteration of fluorophor concentration can be depicted as alteration of signal intensity. Due to hepatic clearance of ICG, FOI signals decay with a time constant of typically three to four minutes. In a pilot study, it was found that the relative FOI signal intensities within one frame do not change significantly after an imaging period of six minutes. Therefore, this duration was determined as the standard examination time.

**Figure 1:**
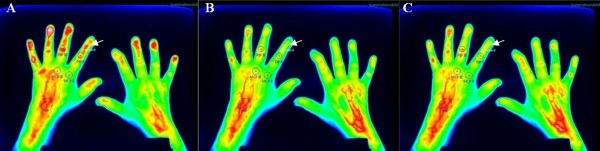
**FOI images of a female RA patient.** Fluorescence optical imaging composite images of the hands of a female patient with rheumatoid arthritis: **(A)** phase 1 (1 to 120 seconds); **(B)** phase 2 (121 to 240 seconds), and **(C)** phase 3 (241 to 360 seconds). Depicted are the placement of the regions of interest for the carpal, metacarpalphalangeal (MCP), and proximal interphalangeal (PIP) joint of the index (II) and middle (III) fingers and the control region at the eponychium of the index finger (white arrow).

FOI findings were analyzed using software provided by the manufacturer (Xiralyze (version 1.0.3), Mivenion GmbH, Berlin, Germany). Electronically generated composite images (CI) were calculated from the mean fluorescence signal intensities of an image stack of sequential subsets of acquired images. In line with two previous publications, we defined CI for three sequential phases with a duration of 120 seconds each (1 to 120 seconds, 121 to 240 seconds, and 241 to 360 seconds) [8,19]. To allow for quantitative assessment, five joints (wrist, MCP II and III, and PIP II and III) of each patient were analyzed using circular regions of interest (ROI), with the size of each ROI defined according to the anatomical size of the corresponding joint (a diameter of 22 pixel for wrists, 12 pixel for MCP- and 10 pixel for PIP-joints; see Figure 1). The resulting fluorescence readout (FLRO) represents, therefore, the mean fluorescence intensity per pixel of the analyzed CI.

To evaluate the influence of the general perfusion of the whole hand on the readout of individual joints, an additional analysis method was developed: the FLRO at the eponychium of the index finger was determined in each patient, an anatomical site not known to be involved in inflammatory processes in patients with RA (using a circular ROI with a diameter of five pixel, placed centrally over the eponychium). In order to standardize individual joint FOI readout results for the general perfusion, we established a fluorescence ratio (FLRA) for each joint by dividing the readout of the joint ROI by the readout of the eponychium of the index finger.

### Statistical analysis

Data evaluation and statistical analyses were performed using SPSS software (version 20, IBM, Ehningen, Germany). We compared the mean FLRO and mean FLRAs of joints with compared with those without evidence of synovitis derived by clinical evaluation (tender or swollen joints) or by established imaging techniques (GSUS, CDUS, MRI) for all three phases and the sum of the three phases using Student’s *t*-test statistic. Statistical significance was concluded when two-sided *P* values were below 0.05.

## Results

**Figure 2:**
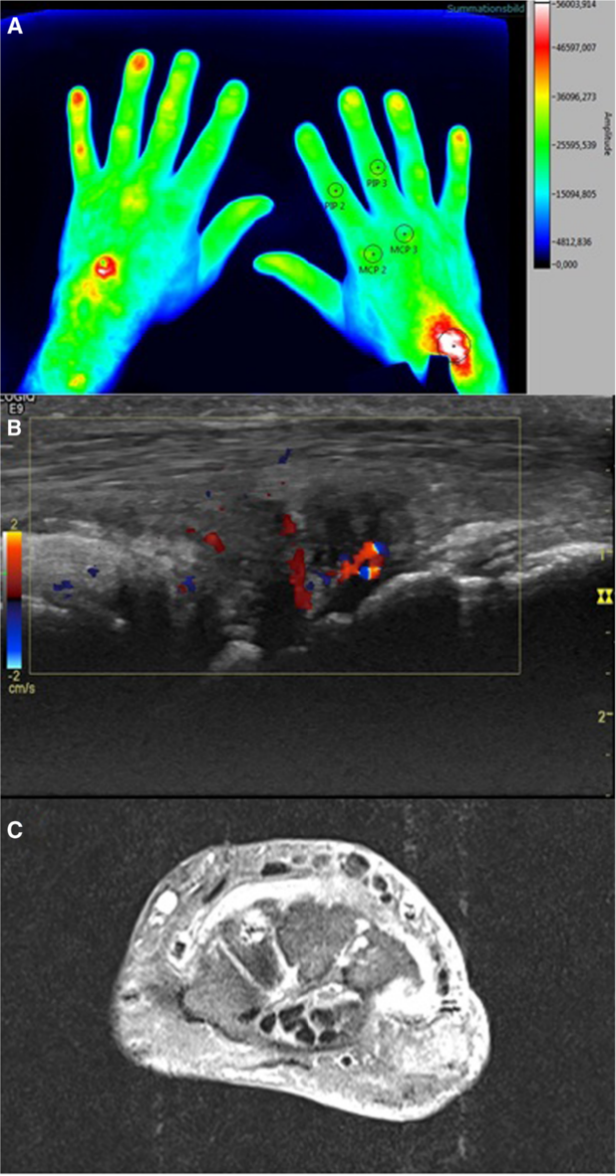
**Correlation of FOI with CDUS and MRI.** Comparison of the fluorescence optical imaging (FOI) composite image **(A)** of phase 2 (121 to 240 seconds) with **(B)** color Doppler ultrasonography (CDUS; showing effusion with hyperperfusion) and **(C)** magnetic resonance imaging (MRI; contrast-enhanced T1-weighted transverse view displaying grade 2 synovitis) of a 50-year-old male patient with carpal arthritis of the right hand.

The study population included 18 subjects, eight women (44%) and 10 men (56%), with a mean (± standard deviation (SD)) age of 63.0 (± 10.0) years. All subjects tolerated the procedure well, and no adverse events were observed. Patient’s clinical and laboratory characteristics, and US and MRI scores are displayed in Table 1. To illustrate the comparison of the employed imaging methods, Figure 2 displays the carpal arthritis of a 50-year-old subject with RA.

**Table 1:** Patient characteristics and results of laboratory and imaging studies^*^

**All patients, n (%)**	**18 (100%)**
Male, n (%)	10 (56%)
Female, n (%)	8 (44%)
Age	63.0 ± 10.0 years
Disease duration	4.9 ± 4.4 years
ESR	25.4 ± 25.3 mm/hour
CRP	17.2 ± 19.6 mg/l
CE, tender joints	7.3 ± 5.0
CE, swollen joints	5.4 ± 5.4
Disease activity (DAS28)	4.6 ± 1.6
Ultrasonography score (US7)	13.8 ± 9.4
MRI score (RAMRIS)	14.6 ± 10.0

*Except where indicated otherwise, values are the mean ± SD. *CE* clinical examination, *CRP* C-reactive protein, *DAS28* disease activity score of 28 joints, *ESR* erythrocyte sedimentation rate, *MRI* magnetic resonance imaging.

A total of 90 joints from the 18 patients were analyzed (18 carpal, 36 MCP, and 36 PIP joints). All 90 joints were evaluated by CE and US and 79 by MRI. The CE revealed 26 of 90 (29%) swollen joints (SJ) and 44 of 90 (49%) tender joints (TJ). The GSUS revealed 39 of 90 (43%), CDUS revealed 23 of 90 (26%) and MRI revealed 39 of 79 (49%) joints that displayed findings consistent with active synovitis. Four of the 79 joints (5%) evaluated by MRI also displayed tenosynovitis of the overlying extensor tendons, but in all four instances the adjoining joint also showed signs of synovitis.

The quantitative analysis for individual joints of the FLRO yielded values ranging from 4.4 to 49.0 × 10^3^ (mean 19.1 ± SD 9.5) for phase 1, 4.2 to 47.9 × 10^3^ (mean 19.6 ± SD 9.2) for phase 2, and 2.6 to 40.9 × 10^3^ (mean 12.8 ± SD 6.8) for phase 3. The results of the quantitative analysis of the FOI data are displayed in Table 2. The comparison yielded significant differences of the mean FLRO between joints with compared to those without evidence of active synovitis determined by US and MRI, but not by CE. Of note, there were significant differences of the mean FLRO between joints with compared to those without synovitis determined by GSUS and MRI for phases 2 and 3, whereas significant differences have been only observed by CDUS in phases 1 and 2.

**Table 2:** **Mean fluorescence readout for joints with compared to those without evidence of synovitis determined by clinical examination or established imaging techniques **^**#**^

		**FOI phase 1 (1 – 120 seconds)**		**FOI phase 2 (121 – 240 seconds)**		**FOI phase 3 (241 – 360 seconds)**	
**Synovitis**	***n***	***mean ± SD***	***P***	***mean ± SD***	***P***	***mean ± SD***	***P***
TJ yes	44	19.1 ± 11.0	ns	19.6 ± 9.0	ns	12.6 ± 5.7	ns
TJ no	46	19.2 ± 7.8		19.6 ± 9.4		13.0 ± 7.8	
SJ yes	26	22.7 ± 12.6	ns	22.3 ± 9.8	ns	14.1 ± 6.2	ns
SJ no	64	17.7 ± 7.5		18.5 ± 8.7		12.3 ± 7.0	
GSUS yes	39	21.0 ± 11.4	ns	23.5 ± 10.4	**	15.8 ± 7.9	**
GSUS no	51	17.7 ± 7.5		16.6 ± 6.8		10.5 ± 4.8	
CDUS yes	23	24.1 ± 12.3	*	23.8 ± 9.3	*	15.0 ± 5.9	ns
CDUS no	67	17.5 ± 7.7		18.2 ± 8.7		12.0 ± 7.0	
MRI yes	39^§^	20.9 ± 10.1	ns	22.3 ± 9.5	*	14.8 ± 7.3	*
MRI no	40^§^	17.8 ± 9.6		17.2 ± 9.2		10.9 ± 6.5	
All	90	19.1 ± 9.4		19.6 ± 9.2		12.8 ± 6.8	

^#^ Fluorescence readout results are displayed for better readability divided by 10^3^; ^§^ 11 joints could not be evaluated with MRI; ^*^P<0.05; ^**^P<0.01.

*CDUS* color Doppler ultrasonography, *FOI* fluorescence optical imaging, *GSUS* grey-scale ultrasonography, *MRI* magnetic resonance imaging; ns, comparison was not significant (*P*≥0.05 with student’s *t*-test), *SD* standard deviation, *SJ* swollen joint by clinical examination, *TJ* tender joint by clinical examination.

To dissect the effect of the overall perfusion of the hand from the perfusion due to active synovitis and to improve FOI results, novel FLRA scores were calculated for each individual joint as described above. The FLRO at the eponychium of the index finger ranged from 8.6 to 42.2 × 10^3^ (mean 20.8 ± SD 9.5) for phase 1, 5.5 to 36.0 × 10^3^ (mean 16.8 ± SD 7.7) for phase 2, and 3.1 to 25.3 × 10^3^ (mean 10.7 ± SD 5.4) for phase 3. The derived values of the FLRAs ranged from 0.37 to 2.27 (mean 0.97 ± SD 0.39) for phase 1, 0.60 to 2.88 (mean 1.20 ± SD 0.39) for phase 2, and 0.62 to 2.87 (mean 1.23 ± SD 0.39) for phase 3. The results of the FLRAs are displayed in Table 3. Compared with the analysis of the FLRO, the FLRA results demonstrated improved discrimination between joints with compared with those without evidence of active synovitis resulting in significant differences of the calculated FLRA values compared with all clinical and imaging techniques in phases 2 and 3. Additionally, analyses comparing FOI with MRI stratified by joint type (carpal, MCP, PIP) were performed (data not shown). Due to the fact that 15 of 18 carpal joints displayed synovitis by MRI, no statistical significant differences were found for FLRO and FLRA for this subgroup analysis. The subgroup analyses of MCP and PIP joints revealed similar findings to the analysis of all joints, with better discrimination of FLRA than FLRO in both subgroups.

**Table 3:** **Mean fluorescence ratios (standardized for overall perfusion of the hand) for joints with compared with those without evidence of synovitis determined by clinical examination or established imaging techniques **^**#**^

		**Ratio FOI phase 1 (1 – 120 seconds)**		**Ratio FOI phase 2 (121 – 240 seconds)**		**Ratio FOI phase 3 (241 – 360 seconds)**	
**Synovitis**	***n***	***Mean ± SD***	***P***	***Mean ± SD***	***P***	***Mean ± SD***	***P***
TJ yes	44	1.02 ± 0.45	ns	1.31 ± 0.48	*	1.33 ± 0.48	*
TJ no	46	0.93 ± 0.33		1.10 ± 0.23		1.13 ± 0.24	
SJ yes	26	1.13 ± 0.50	*	1.43 ± 0.54	**	1.45 ± 0.54	**
SJ no	64	0.91 ± 0.33		1.11 ± 0.26		1.14 ± 0.26	
GSUS yes	39	1.06 ± 0.47	ns	1.33 ± 0.48	*	1.37 ± 0.48	**
GSUS no	51	0.91 ± 0.32		1.10 ± 0.26		1.12 ± 0.26	
CDUS yes	23	1.22 ± 0.46	**	1.52 ± 0.52	**	1.53 ± 0.53	**
CDUS no	67	0.89 ± 0.33		1.09 ± 0.26		1.12 ± 0.26	
MRI yes	39^§^	1.11 ± 0.46	**	1.38 ± 0.47	***	1.41 ± 0.46	***
MRI no	40^§^	0.86 ± 0.33		1.06 ± 0.26		1.08 ± 0.26	
All	90	0.97 ± 0.39		1.20 ± 0.39		1.22 ± 0.39	

^#^ Fluorescence ratios were derived by dividing the readout of each joint by the readout of the control area of the eponychium of the index finger; ^§^ 11 joints could not be evaluated with MRI; ^*^P<0.05; ^**^P<0.01; ^***^P<0.001.

*CDUS,* color Doppler ultrasonography; *FOI,* fluorescence optical imaging; *GSUS,* grey-scale ultrasonography; *MRI,* magnetic resonance imaging; *ns,* comparison was not significant (*P*≥0.05 with student’s *t*-test); SD, standard deviation; *SJ,* swollen joint by clinical examination; *TJ*, tender joint by clinical examination.

A receiver-operating characteristic analysis, comparing the FLRAs of phase 1, phase 2, and phase 3 to detect synovitis confirmed by MRI as gold standard revealed the highest area under the curve for phase 3 (0.67), whereas lower values have been observed for phase 1 (0.58) and phase 2 (0.65). Using a cut-off value for the FLRA of phase 3 of more than 1.2 to detect MRI-confirmed synovitis with FOI, a sensitivity of 26 of 39 (67%; 95% confidence interval (CI), 51 to 79%) and a specificity of 31 of 40 (77%; 95% CI, 62 to 88%) were calculated, with a sensitivity of 21 of 32 (66%; 95% CI, 48 to 80%) for grade 1, and five of seven (71%; 95% CI, 36 to 92%) for grade 2 and 3 MRI-detected synovitis. A stratified analysis by joint type utilizing the same cut-off for FLRA of phase 3 to detect synovitis confirmed by MRI revealed a sensitivity/specificity of 87% (95% CI, 62 to 96%)/0% (95% CI, 0 to 56%) for carpal joints, 42% (95% CI, 23 to 62%)/93% (95% CI, 70 to 99%) for MCP joints, and 100% (95% CI, 57 to 100%)/77% (95% CI, 57 to 90%) for PIP joints.

## Discussion

FOI with the Xiralite system is an emerging imaging technology. However, so far only semiquantitative analyses of FOI results have been reported limiting the strength and reliability of this method. Therefore, we aimed to establish a novel scoring system to allow for a quantitative FOI analysis: introducing and defining circular ROIs for five joints (wrist, MCP II and III, and PIP II and III) FLROs could be calculated representing the mean fluorescence intensity per pixel of the analyzed CI. In order to standardize the quantitative analysis for each patient individually, the FLRO at the eponychium of the index finger has been determined in each patient as a marker of the general perfusion. Dividing the readout of the joint ROI by the readout of the eponychium of the index finger, FLRAs could be calculated for each joint allowing quantitative analysis of FOI results in each patient individually. To validate the novel quantitative FOI scores, other imaging techniques (GSUS, CDUS, and MRI) as well as CE were applied to compare differentiation of joints with and joints without evidence of synovitis. To our knowledge this is the first study evaluating the ability of this novel imaging modality comparing quantitative fluorescence readout to synovitis detected by MRI and US.

In arthritic conditions, angiogenesis is highly dysregulated [33]. Furthermore, hypervascularisation and angiogenesis of the synovial membrane are a hallmark in RA patients [34] and strongly linked to bone destruction. The degree of synovial vascularisation correlates well with the disease activity of a given joint [35,36], as well as radiographic progression [37] and with the therapeutic response in patients with RA [38]. Therefore, FOI might be a useful tool to detect and monitor disease activity in RA patients by visualization of microperfusion changes in affected joints.

Using the quantitative approach for the evaluation of the obtained FOI results, significant differences of the mean FLRO have been observed between synovitic and non-synovitic joints for the results of phases 2 and 3, which were even more striking after standardization by calculating the FLRAs. Unfortunately, no definite cut-off values for perfect separation of synovitic from non-synovitic joints could be established in our pilot study. However, a sensitivity of 67% and a specificity of 77% was calculated using a cut-off value for the FLRAs of phase 3 of more than 1.2 to detect MRI-confirmed synovitis with FOI, which demonstrates the diagnostic yield of this novel imaging method. The subgroup analyses stratified by joint type revealed for this cut-off a higher sensitivity for carpal and PIP joints than for MCP joints. Larger future studies should address if different joint types deserve distinct cut-offs for FLRA interpretation.

In each FOI sequence, three phases were scaled, giving different results in comparison to the other imaging modalities. In the publication by Werner et al. [8] phase 1 displayed the highest agreement with CE and CDUS, whereas the highest sensitivity was observed in phase 2, comparable with the sensitivity of US in relation to MRI. Using MRI as standard of reference, the FOI system displayed a sensitivity of 76% and a specificity of 54% using different analysis parameters. Consistent to these findings, a sensitivity of 67% and a specificity of 77% were calculated for FOI in our study compared with MRI findings. With regard to the different phases analyzed, our results differ partially to the previous observations, as phase 1 did not show similar significant results, whereas phases 2 and 3 correlated best with all imaging modalities following standardization.

In a recent study by Meier et al. [19], FOI was compared with CE and three T-weighted MRI. Using MRI as standard of reference, FOI displayed a sensitivity of 39.6% and a specificity of 85.2%. In that study, the diagnostic accuracy of the FOI was altogether lower and particularly limited in mild synovitis, whereas it performed substantially better in severely inflamed joints.

So far, different methods for interpreting the FOI results have been deployed [8,19,28]. Werner et al. [8] used a semiquantitative score, where the signal enhancement has been graded in percentages. In contrast, Meier et al. [19] utilized a semiquantitative assessment where synovitis was graded in each joint ranging from 0 to 3. As outlined by these investigators, a semiquantitative approach revealed only moderate interreader agreement with poor findings for the PIP and DIP joints, respectively [19]. Although we did not test for interreader agreement formally, our quantitative approach using directly computed FLRO and FLRA circumvents these problems of semiquantitative analyses.

In their study, Dziekan et al. used a ROI placed over the corresponding finger nail to standardize the FLRO of each finger joint to the interpersonal changes of overall perfusion [28]. Their quantitative analyses were not stratified into the three phases, as employed in our and other studies [8,19,28]. The authors noted that with their proposed method of quantitative FOI analysis a good separation between asymptomatic and inflamed joints (delineated by clinical examination of patients with RA) was not possible. They mainly attributed that finding to the lack of a ‘gold standard’ , because no other imaging methods (e.g. US or MRI) were employed in their study.

Werner et al. utilized the percentage of surface area of the affected joint to arrive at a ordinal scale from 0 to 3, whereas the publication by Meier et al. used the criteria of the RAMRIS semiquantitative scoring system [32], developed to evaluate synovitis detected by MRI, also for semiquantitative interpretation of the FOI data [8,19]. Both approaches are based on changes of fluorescence intensity of the joint of interest compared with the fluorescence in unaffected joints over time, and are therefore not prone to be affected strongly by the background change of increasing and then decreasing concentrations of ICG. This difference of ICG concentrations during the three phases may explain why FLRA displayed a better discrimination between inflamed and non-inflamed joints then the plain FLRO results.

We are aware of some limitations concerning the image interpretation and quantification of pathological changes. Although the FOI procedure itself is standardized, consistent standards for image adjustment and automated interpretation are not yet established. We have chosen to set ROIs over those finger joints, which are typically involved in RA. This allows for better quantitative examination, but each ROI has to be set manually and the radius of the different ROIs needs still to be more accurately defined.

## Conclusions

Our pilot study demonstrates that a quantitative assessment of the fluorescence results obtained by FOI is feasible, although so far no definite cut-off value could be delineated that perfectly separates inflamed from non-inflamed joints in patients with RA. Based on the better discrimination of FLRA compared with FLRO we postulate to use FLRA in larger studies to further set and evaluate the diagnostic yield of quantitative interpretation of FOI in the clinical care of patients with suspected or established RA.

## Abbreviations

Anti-CCP antibodies: Anti-cyclic citrullinated peptide antibodies; CDUS: Color Doppler ultrasonography; CE: Clinical examination; CRP: C-reactive protein; DAS 28: Disease activity score of 28 joints; ESR: Erythrocyte sedimentation rate; FLRA: Fluorescence ratio; FLRO: Quantitative fluorescence readout; FOI: Fluorescence optical imaging; FOV: Field of view; GSUS: Grey-scale ultrasonography; ICG: Indocyanine green; MCP: Metacarpophalangeal; MRI: Magnetic resonance imaging; MTP: Metatarsophalangeal; PIP: Proximal interphalangeal; RA: Rheumatoid arthritis; RF: Rheumatoid factor; ROI: Regions of interest; SD: Standard deviation; SJ: Swollen joint; ST: Slice thickness; TE: Echo time; TJ: Tender joint; TR: Repetition time; US: Ultrasonography.

## Competing interests

JB is employed by the manufacturer of the system and software used to perform FOI in this study. All other authors declare no conflict of interest.

## Authors’ contributions

VS, WH, MF, and BE designed the study. VS and WH carried out the acquisition and quantification of clinical examination, sonography, and FOI data. PH graded the MRI examinations. JB contributed to the utilization of the FOI quantification software. VS and BE performed the statistical analysis. VS, WH, PH, CS, MM, MF, and BE analyzed and interpreted the data and prepared the manuscript. All authors read and approved the final manuscript.
